# Ethnic-specific associations of rare and low-frequency DNA sequence variants with asthma

**DOI:** 10.1038/ncomms6965

**Published:** 2015-01-16

**Authors:** Catherine Igartua, Rachel A. Myers, Rasika A. Mathias, Maria Pino-Yanes, Celeste Eng, Penelope E. Graves, Albert M. Levin, Blanca E. Del-Rio-Navarro, Daniel J. Jackson, Oren E. Livne, Nicholas Rafaels, Christopher K. Edlund, James J. Yang, Scott Huntsman, Muhammad T. Salam, Isabelle Romieu, Raphael Mourad, James E. Gern, Robert F. Lemanske, Annah Wyss, Jane A. Hoppin, Kathleen C. Barnes, Esteban G. Burchard, W. James Gauderman, Fernando D. Martinez, Benjamin A. Raby, Scott T. Weiss, L. Keoki Williams, Stephanie J. London, Frank D. Gilliland, Dan L. Nicolae, Carole Ober

**Affiliations:** 1 Department of Human Genetics, University of Chicago, 920 East 58th Street, CLSC 425, Chicago, Illinois 60637, USA; 2 Department of Medicine, Johns Hopkins University, Baltimore, Maryland 21224, USA; 3 Department of Medicine, University of California San Francisco, San Francisco, California 94143, USA; 4 CIBER de Enfermedades Respiratorias, Instituto de Salud Carlos III, Madrid 28029, Spain; 5 Arizona Respiratory Center and BIO5 Institute, University of Arizona, Tucson, Arizona 85721, USA; 6 Department of Public Health Science, Henry Ford Health System, Detroit, Michigan 48202, USA; 7 Hospital Infantil de Mexico Federico Gomez, Mexico City 06720, Mexico; 8 Department of Pediatrics, University of Wisconsin School of Medicine and Public Health, Madison, Wisconsin 53726, USA; 9 Department of Epidemiology, Johns Hopkins University, Baltimore, Maryland 21224, USA; 10 Department of Preventive Medicine, Keck School of Medicine, University of Southern California, Los Angeles, California 90033, USA; 11 School of Nursing, University of Michigan, Ann Arbor, Michigan 48202, USA; 12 International Agency for Research on Cancer, Lyon 69372, France; 13 Department of Internal Medicine, University of Wisconsin School of Medicine and Public Health, Madison, Wisconsin 53726, USA; 14 Department of Medicine, University of Wisconsin School of Medicine and Public Health, Madison, Wisconsin 53726, USA; 15 Division of Intramural Research, Department of Health and Human Services, National Institute of Environmental Health Sciences, National Institutes of Health, Research Triangle Park, North Carolina 27709, USA; 16 Department of Biological Sciences, North Carolina State University, Raleigh, North Carolina 27607, USA; 17 Department of Bioengineering and Therapeutic Sciences, University of California San Francisco, San Francisco, California 94143, USA; 18 Channing Division of Network Medicine, Harvard Medical School, Boston, Massachusetts 2115, USA; 19 Division of Pulmonary and Critical Care Medicine, Harvard Medical School, Boston, Massachusetts 2115, USA; 20 Center for Health Policy and Health Services Research, Henry Ford Health System, Detroit, Michigan 48202, USA; 21 Department of Internal Medicine, Henry Ford Health System, Detroit, Michigan 48202, USA; 22 Departments of Medicine and Statistics, University of Chicago, Chicago, Illinois 60637, USA

## Abstract

Common variants at many loci have been robustly associated with asthma but explain little of the overall genetic risk. Here we investigate the role of rare (<1%) and low-frequency (1–5%) variants using the Illumina HumanExome BeadChip array in 4,794 asthma cases, 4,707 non-asthmatic controls and 590 case–parent trios representing European Americans, African Americans/African Caribbeans and Latinos. Our study reveals one low-frequency missense mutation in the *GRASP* gene that is associated with asthma in the Latino sample (*P*=4.31 × 10^−6^; OR=1.25; MAF=1.21%) and two genes harbouring functional variants that are associated with asthma in a gene-based analysis: *GSDMB* at the 17q12–21 asthma locus in the Latino and combined samples (*P*=7.81 × 10^−8^ and 4.09 × 10^−8^, respectively) and *MTHFR* in the African ancestry sample (*P*=1.72 × 10^−6^). Our results suggest that associations with rare and low-frequency variants are ethnic specific and not likely to explain a significant proportion of the ‘missing heritability’ of asthma.

## Abstract (web summary)

Common variants account for only a small amount of the heritable risk for developing asthma. Using a meta-analysis approach, Igartua *et al*. identify one low-frequency missense mutation and two genes with functional variants that are associated with asthma, but only in specific ethnic groups.

## Graphical abstract

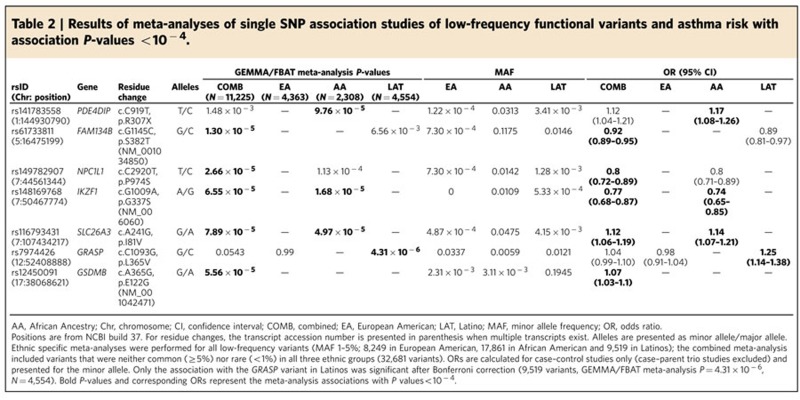

Asthma is a common, chronic inflammatory disease of the airways with typical onset in childhood. Heritability estimates indicate that approximately half the variation in risk is attributable to genetic factors^1^,^2^,^3^; yet, the common variants identified by genome-wide association studies (GWAS) account for very little of the genetic risk^4^,^5^,^6^,^7^. This so-called ‘missing heritability’ in asthma and other common diseases has been attributed to many potential causes that generally fall into two categories^8^. First, the genetic variants interrogated by GWAS may not capture all relevant variation. In particular, rare variants, which comprise the bulk of genetic variation in the human genome and are predicted to have larger phenotypic effects than common variants, are not represented or tagged by the single-nucleotide polymorphisms (SNPs) that are included on the genotyping arrays typically used in GWAS. Second, the statistical approaches used in GWAS may be overly simplistic and not adequately model the genetic architecture of asthma, which is probably polygenic and includes many gene–environment interactions^9^.

In this study, we begin to address the first limitation of GWAS by investigating the role of rare (minor allele frequency (MAF) <1%) and low-frequency (MAF 1–5%) variants in asthma. We conducted this meta-analysis study in the largest ethnically diverse sample of asthma cases, non-asthmatic controls and case–parent trios, which include 13 studies^4^,^10^,^11^ from 8 groups of investigators who compose the EVE Consortium on Asthma Genetics^4^,^10^. We conducted analyses separately in each of the 13 study samples and used meta-analyses to first combine results within each of three major ethnic/racial groups (European American, African American/African Caribbean and Latino) and then across all ethnic/racial groups to yield measures of association for the combined sample. Here we report associations with a low-frequency functional variant and lower risk for asthma in Latinos in a novel gene, *GRASP*, and with functional variants in the *MTHFR* gene in an African ancestry sample. We also report an association in Latinos and in the combined sample with functional variants in *GSDMB*, a gene at the 17q12–21 asthma locus^4^,^5^,^12^,^13^,^14^, which is attributed to a putatively damaging common missense mutation.

## Results

### Single-variant association analyses

We first evaluated the individual effects of low-frequency, putatively functional exonic variants on asthma risk in 11,225 subjects representing European Americans, African Americans, African Caribbeans, Mexican Americans, Mexicans and Puerto Ricans (Table 1). We tested each low-frequency variant, defined as having a MAF between 1% and 5% within each ethnic group. For the meta-analysis in the combined sample, we excluded variants that were either common (MAF ≥5%) in all three ethnic groups or rare (<1%) in all three ethnic groups (Supplementary Table 1 and Supplementary Table 2). This definition allows us to include SNPs that may be common in one population but rare in the other(s). The quantile–quantile plots for the analyses of low-frequency variants indicated that there was no inflation of test statistics (Supplementary Fig. 1). One missense mutation (c.C1093G, p.L365V) in the *GRASP* gene was associated with asthma risk in the Latinos after correcting for multiple testing (9,519 tests; Genome-wide Efficient Mixed Model Association (GEMMA)^15^/Family Based Association Test (FBAT)^16^ meta-analysis, *P*=4.31 × 10^−6^; *N*=4,554; odds ratio (OR)=1.25; MAF=0.012). Six additional low-frequency variants were modestly associated at suggestive levels of significance (*P*<10^−4^) in at least one of the three groups or in the combined sample (Table 2). Of these, five missense mutations (one each in *FAM134B*, *NPC1L1*, *IKZF1*, *SLC26A3* and *GSDMB*) showed evidence of association with asthma in the combined sample, two of which (*IKZF1* and *SLC26A3*) were more significant in the African ancestry sample. The low-frequency variants at *FAM134B* (c.G1145C, p.S382T; transcript accession NM_001034850), *NPC1L1* (c.C2920T, p.P974S) and *IKZF1* (c.G1009A, p.G337S; transcript accession NM_006060) were all associated with lower risk for asthma, whereas the variants in *SLC26A3* (c.A241G, p.I81V) and *GSDMB (*c.A365G, p.E122G; transcript accession NM_001042471) were associated with increased risk for asthma. An additional nonsense mutation (c.C919T, p.R307X) in *PDE4DIP* was also associated with asthma risk in the African ancestry sample. The latter result is noteworthy for two reasons. First, an exome-sequencing study in a European American, non-Hispanic white nuclear family with two asthmatic and two non-asthmatic children also reported a missense mutation in *PDE4DIP* (c.A907C, p.I303L) segregating with asthma^17^. Second, this gene encodes a protein that interacts with *PDE4D*, a gene that was identified in a GWAS of asthma^18^. Our results further implicate this pathway in asthma pathogenesis.

The missense mutation in *GSDMB* is also intriguing, because this gene is at the well-replicated 17q12–21 asthma locus^4^,^5^; this signal is largely attributable to the GALA II Mexican American sample (GEMMA, *P*=1.1 × 10^−4^; *N*=1,172). This variant showed striking MAF differences between the groups, ranging from 0.19 in the Latinos to 0.0031 in the African Americans and 0.0023 in the European Americans, but was included because it was neither rare nor common in all three groups (see Methods).

### Conditional analyses at the 17q12–21 locus

To determine whether the association with this variant (c.A365G, p.E122G; rs12450091) in Latinos and the combined sample represents independent risk for asthma or is in linkage disequilibrium (LD) with the common variants at the 17q12–21 locus that have been associated with asthma in previous GWAS^4^,^5^,^19^,^20^, we performed conditional analyses of rs12450091 in the case–control samples only (case–parent trio studies were excluded and meta *P*-value recalculated: Latino GEMMA meta-analysis *P*=6.89 × 10^−5^, *N*=3,912; combined GEMMA meta-analysis *P*=5.52 × 10^−5^, *N*=9,501). For this analysis, we first identified three common variants (one synonymous, one missense and one intronic) at this locus that were included on the exome array and associated with asthma in previous reports (G at rs907092 (refs 4, 5, 19), G at rs2305480 (refs 5, 20) and T at rs7216389 (refs 5, 21, 22)). In Latinos, the risk allele at rs12450091 (C) tags 28% of the high-risk *GGT* haplotypes (*GGCT* frequency 18.09%, *GGTT* frequency 45.84%) and is not in LD with any of the known expression quantitative trait loci reported at this locus^12^,^21^,^22^,^23^,^24^. We then repeated the association studies with rs12450091 (c.A365G, p.E122G) in *GSDMB* in three separate analyses, including each of the common variants as a covariate in each analysis to ‘remove’ the effects of the common variant on the association signal. In each of these three analyses, the evidence for association between rs12450091 and asthma was reduced from *P*=6.89 × 10^−5^ to *P*≤0.022 in Latinos and from *P*=5.52 × 10^−5^ to *P*≤0.019 in the combined sample (Table 3). These results suggest that although most of the evidence for association with this variant was shared with the associated common variants, the residual signal in conditional analyses indicates that this missense substitution or a variant in strong LD with the missense substitution contributes additional, independent risk to asthma in Latinos.

### Power analyses for low-frequency variants

After testing for nearly 33,000 functional low-frequency exonic variants in 11,225 subjects, only 1 variant remained statistically significant in Latinos after correcting for testing of 9,519 of variants and only 6 were modestly associated at *P*-values <10^−4^ in the African ancestry or the combined sample. To assess whether the paucity of associations was due to lack of power, we performed simulations to determine the power of our studies to detect associations with asthma for low-frequency variants. We considered ORs of 1.5, 2, 2.5, 3 and 4, within the expected ranges of effect sizes for low-frequency variants with moderate-to-high effects. In fact, we had at least 80% power to detect associations with variants with frequencies ≥1% and ORs ≥2.5 in the combined sample. In the European American and Latino samples, we had 80% power to detect variants with frequencies ≥1% and ORs ≥4. We had lowest power in the African ancestry sample, in which there was 80% power to detect variants with frequencies >2% and ORs ≥4 (Supplementary Fig. 2). Thus, the lack of significant associations with low-frequency variants in our study could be due to low power in the ethnic/racial groups, small effect sizes of variants in this frequency range, or both.

### Gene-based analyses

Studies of rare variants have virtually no power to detect individual associations in samples of even many thousands of individuals^25^,^26^,^27^. Rather, gene-based tests that combine the evidence for association among all variants within each gene provide more power to detect associations. To test the hypothesis that the overall burden of functional variants at each gene confers risk for asthma, we performed a gene-based association test as implemented in MetaSKAT-O^28^ for each of the 10,208 genes represented on the exome array that had three or more functional variants present in at least two case–control samples. We included in these analyses all putatively functional variants on the array regardless of allele frequency. We performed two sets of analyses. In the first we weighted all variants equally, and in the second we gave larger weights to rarer variants (MetaSKAT-O default). For each of those analyses, we considered all potentially functional variants (missense, nonsense and splice site) and then considered only variants predicted to be ‘probably damaging’ by Polymorphism Phenotyping v2 (PolyPhen-2)^29^. In the analysis with equal weights and defining functional variants more broadly, associations with *GSDMB* and *ZPBP2*, two genes at the 17q12–21 asthma locus, were significant in the combined (MetaSKAT-O: *P*=6.10 × 10^−10^, 16 variants and *P*=1.34 × 10^−6^, 7 variants, respectively; *N*=9,501) and Latino (MetaSKAT-O: *P*=1.02 × 10^−6^, 12 variants and *P*=1.73 × 10^−6^, 7 variants, respectively; *N*=3,912) samples after Bonferroni correction for 9,534 and 10,439 genes, respectively. In addition, the *MTHFR* gene was significant (MetaSKAT-O: *P*=1.72 × 10^−6^, 11 variants; *N*=2,308) in the African ancestry sample after multiple testing correction for 10,342 genes. When we considered only variants with PolyPhen-2 prediction of ‘probably damaging’, the association with *GSDMB* remained significant (combined MetaSKAT-O: *P*=4.09 × 10^−8^, 7 variants, *N*=9,501; Latino MetaSKAT-O: *P*=7.81 × 10^−8^, 5 variants, *N*=3,912), but the associations with the *ZBPB2* and *MTHFR* genes did not. No genes were significant when larger weights were applied to rare variants in either of the analyses (Table 4, Supplementary Table 3, Supplementary Figs 3–6), suggesting that these gene-based associations are driven largely by the more common functional variants that are present on the array. In fact, when we remove one common missense SNP in *GSDMB* (c.G844A, p.G282R; rs2305479; MAF 0.335 in Latinos), the evidence for association of this gene in the test that considered only PolyPhen-2 predicted damaging variants becomes nonsignificant (equal weights; MetaSKAT-O *P*=1.0 in Latinos, *N*=3,912 and *P*=0.276 in the combined sample, *N*=9,501). Similar to *GSDMB*, the associations with *ZPBP2* were no longer significant after removing the one common missense variant (c.G518T p.S173I; rs11557467; MAF 0.3576 in Latinos) and testing the association of the remaining six variants in the Latino (MetaSKAT-O: *P*=0.54, *N*=3,912) and the combined sample (MetaSKAT-O: *P*=0.12, *N*=9,501). This suggests that the association with *ZPBP2* is driven by this common variant. In contrast, removing the one common missense variant in *MTHFR* (c.C665T p.A222V; rs1801133; MAF=0.11 in African ancestry) does not eliminate the gene-based association signal (nine rare, one low-frequency variant; MetaSKAT-O: *P*=0.002, *N*=2,308), indicating that the association with variants in the *MTHFR* gene is attributable to common, low-frequency and rare variants.

### Assessing coverage of exonic variation on the exome array

Lastly, we assessed how well the variants on the exome array captured the low-frequency and rare functional variation present in the genomes of asthmatic individuals. We had available whole-genome sequences (WGS) for 278 asthmatics (101 European Americans, 93 African Americans and 84 Latinos) as part of ongoing studies of the EVE Consortium^4^,^10^, including 172 of the individuals in this study (43 European Americans, 44 African Americans and 85 Latinos). We selected all functional exonic variants with MAF<5% in any of the three groups from the WGS and compared the overlap between the missense, nonsense and splice site mutations present in the WGS with the variants on the exome array. Overall, there were 105,943 rare or low-frequency variants in 16,359 genes in the WGS of the 278 asthmatic individuals, of which 65,170 (61.51%) variants in 14,222 genes (86.93%) were on the array (Table 5). Surprisingly, the proportion of functional variants on the array was similar in the three ethnic groups (range: 62.77% to 63.87%), despite the fact that European Americans were the predominant source of variation in the array design (~9,000 of ~12,000 subjects)^30^. Finally, we compared the number of genes in the WGS with functional exonic variants to the number of genes represented on the exome array. For this analysis, we stratified the 16,359 genes in the WGS with functional exonic variation into three groups: (i) genes not represented on the array; (ii) genes represented on the array, but not all variants are included; and (iii) genes represented on the array and all functional variants are included. Overall, 2,137 genes (13.06%) were not represented on the array and those genes included 5,283 functional exonic variants in the WGS. An additional 10,686 genes (65.32%) had incomplete sets of functional variants, with 35,490 variants present in the WGS but absent on the array. Finally, 3,536 genes (21.61%) had all 9,370 functional exonic variants present in the WGS of asthmatic individuals (Supplementary Table 4). At the 17q12–21 locus, *ORMDL3* had one rare missense mutation in Latinos (c. C287T, p.T96M; rs200735199; MAF 0.005) in the WGS and it was not included on the array, *GSDMB* had eight variants in the WGS and seven were on the array (one missense mutation missing, c.C689T, p.S230F; transcript accession NM_001165959; rs183724236; MAF 0.005 in African Americans), *IKZF3* had one missense variant in the WGS and it was on the array, and *ZPBP2* had five variants in the WGS and four were on the array (one missense mutation missing, c.G586T, pG196C; transcript accession NM_198844; rs371561983; MAF 0.005 in Latinos). Thus, overall, the rare and low-frequency functional variation at the 17q12–21 locus was reasonably well covered by the array.

## Discussion

Our study is the largest to date, to assess the effects of rare variants on asthma risk in ethnically diverse individuals. Although we were limited by the variation present on the array and by relatively small samples within each ethnic/racial group, our results provide several insights into the genetic architecture of asthma. First, associations with rare or low-frequency variants are likely to be ethnic specific. The few significant associations that we detected were all limited to a single ethnic group, despite having the greatest power to detect associations in the combined sample. Two novel associations, one with a missense mutation in the *GRASP* gene in Latinos and one with the many rare missense mutations in the *MTHFR* gene in African Americans/African Caribbeans, were revealed. The *MTHFR* gene harbours 11 missense mutations in African Americans, only one of which is common (rs1801133), and showed a gene-level association with asthma. Although most mutations in this gene were not predicted to be ‘damaging’ by PolyPhen-2 scores, the sheer number of potentially functional variants and the strong association with asthma in the African ancestry sample makes this gene a good candidate for further studies. Common variants in *MTHFR*, encoding methylenetetrahydrofolate reductase, have been associated with many common conditions, including neural tube defects, vascular disease and pregnancy complications, among others^18^,^31^,^32^,^33^. There have been conflicting reports as to whether the common C677T variant in this gene is associated with asthma or allergic diseases in European populations^4^,^5^,^34^,^35^,^36^, although maternal folate levels in pregnancy have been associated with risk of early asthma outcomes in children^37^,^38^. The missense mutation in *GRASP* that is associated with asthma in Latinos occurs at a frequency of 0.012 in this population, at higher frequency (0.034) in European Americans and very low-frequency (0.006) in the African ancestry samples. The fact that the association was confined to Latinos even though we had greater power to detect associations in the European Americans suggests that the effects of this variant on asthma risk might involve interactions with other variants or environmental exposures that are more common in Latinos, but this single-variant association requires replication in an independent Latino sample or functional validation of this variant’s role in asthma risk. *GRASP* encodes GRP1 (general receptor for phosphoinositides-1)-associated scaffold protein and has been shown to be highly expressed in the brain with lower levels of expression in the lung, heart, embryo, kidney and ovary^39^. Although little is known about the function of this gene or its potential role in asthma, scaffold proteins have been implicated in asthma signalling pathways^40^,^41^.

Second, among the six low-frequency variants showing suggestive evidence for association with asthma (Table 2), three variants were associated with decreased risk and three were associated with increased risk. The discovery of potentially protective alleles in three genes, *FAM134B* (family with sequence similarity 134, member B1), *NPC1L1* (Niemann-Pick disease, type C1, gene-like 1) and *IKZF1* (IKAROS family zinc finger 1), is intriguing because elucidating the function of these variants could identify novel therapeutic targets for asthma.

Third, the *GSDMB* (gasdermin B) locus on chromosome 17q12–21 is within an LD block that has been robustly associated with asthma^4^,^5^,^6^,^12^,^13^; yet, the causal gene and its function are still unclear. The SNPs discovered by GWAS that are associated with asthma are also associated with the expression of both *GSDMB* and its close neighbour *ORMDL3* in white blood cells^12^, peripheral blood mononuclear cells^21^, lymphoblastic cells lines^42^, CD4+ T cells^23^,^24^ and lung tissue^22^, and it has been assumed both that the causal SNPs at this locus are expression quantitative trait loci for these genes and that one or both of these genes are involved in asthma pathogenesis. Our study revealed several lines of evidence of association with *GSDMB* and extensive functional variation within this gene, including a common missense mutation rs2305479 (c.G871A, p.G291R; rs2305479) that is predicted to be ‘probably damaging’ by PolyPhen-2. This common variant was identified in the previous EVE meta-analysis of GWAS^4^ (*P*=1.21 × 10^−12^ in the combined sample) and is in strong LD with the GWAS-identified SNP, but its potential as a causal candidate has not previously been discussed. Our study suggests rs2305479 as a good candidate for the causal variant at this important locus and implicates *GSDMB* more directly in asthma risk. We identified a second potential causal variant, a common SNP within a splicing site in *GSDMB* (rs11078928; c.661-2A>G; transcript accession NM_001165959). This SNP was previously shown to have a genotype-dependent effect on *GSDMB* transcript levels, whereby the asthma risk allele (A) was associated with increased expression of an alternate transcript lacking exons 5–8 (ref. 43). Although not much is known about the function of *GSDMB* or of its alternative splice forms, other gasdermin genes are involved in apoptosis in epithelial cells^44^, processes and cells that are relevant to asthma. It is additionally notable that in contrast to *GSDMB*, there was only one rare missense mutation in *ORMDL3* in Latinos (rs200735199, c457T, p.T96M; transcript accession NP_644809; MAF 0.005) in the WGS from 278 asthmatics, and this variant was not included on the exome array.

In summary, we report significant associations of asthma with rare or low-frequency variants in two novel genes, *GRASP* and *MTHFR*, in Latinos and the African ancestry sample, respectively, and with a potentially damaging common missense mutation in *GSDMB* in Latinos and the combined sample. Based on the WGS from 278 asthmatic subjects, our study included over 60% of all putatively functional rare and low-frequency variation. Thus, even if we discovered only half of all truly associated variants, it is unlikely that rare and low-frequency variants will account for a significant portion of the heritability of asthma.

## Methods

### Study subjects

The subjects in the study were from 13 individual studies from eight investigators that comprise the EVE Consortium on the Genetics of Asthma. This sample has large overlap with our previous asthma meta-analyses^4^,^10^, but includes three additional studies: GALA II, COAST and ALHS. Descriptions of each of the studies can be found in prior published work^4^,^10^,^11^ or in Supplementary Note 1. The results reported here are based on analyses of 4,794 asthma cases, 4,707 asthma controls and 590 case–parent trios (Table 1). All participants, or the parents of minors, provided written informed consent. The relevant local institutional research ethics committee approved each contributing study. Albert Einstein College of Medicine of Yeshiva University, Baylor College of Medicine, Brigham and Women's Hospital, Children's Memorial Hospital, Columbia University, Henry Ford Health System, Johns Hopkins University, Kaiser Permante, NIEHS (USA), National Institute of Public Health, Mexico (NIPH), Northwestern University, San Juan VA Medical Center, University of California San Francisco, University of Chicago, University of Southern California, University of Wisconsin Madison, WIRB for Torre Medica Auxilio Mutuo.

### Exome array

The Illlumina Infinium HumanExome BeadChip genotyping array interrogates 247,870 variants discovered from the exome and genome sequences of ~12,000 individuals of European American, African American, Latino and Asian subjects from a range of common disorders. Variants included on the array are predicted to have a functional effect on the protein and are enriched for missense mutations, nonsense mutations and variants within predicted splice sites, as previously described^30^.

### Genotyping

A total of 7,879 samples from 7 studies (CAG, CAMP, COAST, GALA II, GRAAD, MCCAS and SAPPHIRE), including 107 blind duplicates, were genotyped at the Northwest Genomics Center at the University of Washington. An additional 2,440 CHS samples were genotyped at the Molecular Genomics Core at the University of Southern California, using a different version of the array, and 1,585 ALHS samples were genotyped at the Center for Inherited Disease Research at Johns Hopkins University. Genotype calling was performed using Genotyping Module version 1.9.4, GenTrain version 1.0 in GenomeStudio version 2011.1 for all projects, except for CHS that used GenTrain version 2.0. Genotype clustering was defined using our study samples and was completed independently for each project.

Because of the different array design used for the CHS samples, the union of 239,909 (96.79%) variants was genotyped in the 13 sample sets. An additional 7,961 variants were genotyped in all the other samples and were subjected to quality control and meta-analysed independent of CHS.

### SNP quality control

Among the variants present on the Illumina HumanExome BeadChip array, 244,794 (98.76%) passed quality control (Supplementary Table 5). We excluded from analyses variants with genotype call rates <95%, Hardy–Weinberg equilibrium *P*-values <10^−4^ in any of the studied populations (African American, African Caribbean, European American and Mexican/Mexican American or Puerto Rican) and 333 caution sites reported as problematic by the exome array design group ( http://genome.sph.umich.edu/wiki/Exome_Chip_Design). Among the variants passing quality control, 197,339 (79.61%) were variable in the individuals under study. In the combined sample, 23,206 (11.76%) variants were common (MAF≥5%) to all ethnic groups, 36,413 (18.45%) were low-frequency (MAF 1–5%) and 137,720 (69.79%) were rare (MAF<1%) in all three groups. Private variants for each group and counts of variants tested are shown in Supplementary Table 1 and Supplementary Table 2. Additional probe quality and genotype-calling evaluations were performed and are described in Supplementary Note 2.

### Sample quality control

Six hundred and seventy-nine subjects were excluded from analysis (Supplementary Table 6), resulting in a final sample size of 11,225 individuals. Sample exclusions included gender discordances, sample duplications, Mendelian errors in trios, as implemented in PLINK^45^, missing case–control status and incomplete trios. In addition, we completed principal component analysis using the R function *prcomp* to identify ancestry outliers (Supplementary Figs 7–9). Principal component analysis was carried out by using the ancestry informative markers present on the exome array and including HapMap^46^ CEU, YRI, CHB, JPT and MEX subjects. Fifty-six samples were excluded because one or both of the first two principle components were more than six s.d. from the mean of the expected ethnic group. These inferred principle components were applied as covariates in the gene-based tests.

### Single variant analyses

We subjected functional low-frequency variants to single variant association analysis for each of the three ethnic groups (European American, African American and Latino) and for the combined sample. Low-frequency variants (1–5%) were included in each of the ethnic-specific meta-analyses (8,249 in the European Americans, 17,861 in the African ancestry and 9,519 in the Latinos) and the variants that were neither common (≥5%) in all 3 ethnic groups nor rare (<1%) in all three ethnic groups (32,681 variants) were meta-analysed in the combined sample (Supplementary Table 2). To control for population stratification and relatedness, single SNP association tests for case–control studies were completed using the linear mixed model GEMMA^15^. For the samples comprising case–parent trios, we used the FBAT^16^. *Z*-score statistics obtained from GEMMA and FBAT were combined in a meta-analysis as a weighted sum to reflect the power of each independent study. The weight (*w*) used for each SNP is a function of allele frequency (*p*), proportion of cases (*v*) and sample size (*N*) 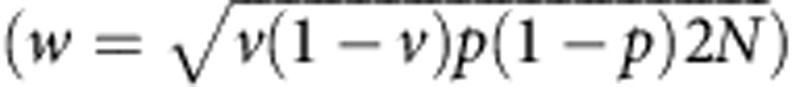
10 of each study. *P*-values were calculated using standard normal approximations and Bonferroni significance thresholds were applied (*α*=0.05) to correct for the number of variants tested within each group and in the combined sample. ORs were obtained for samples analysed with GEMMA as a linear combination of log ORs with weights proportional to the s.e. of the log ORs using the R package *meta*.

### Power analyses for single-variant analyses

For each ethnic group and for the combined sample, we performed the following simulations. We randomly selected 1,000 variants with MAF between 0.5% and 5%, and simulated genotypes 1,000 times for each of 5 ORs (OR=1.5, 2, 2.5, 3 and 4). We used the same study structure (sample sizes and case–control or parent offspring designs) and observed MAF distributions that were present in our data. *Z*-scores were then meta-analysed with the weighted approach described in the single-variant meta-analysis methods and power was calculated with a predefined *α* of 0.05. We plotted power estimates versus MAF and implemented a local linear regression fit with the R function *loess* (Supplementary Fig. 2).

### Gene-based analyses

Functional variants sets were defined two ways: (1) missense, nonsense or variants within splice sites or (2) coding variants with PolyPhen-2 (ref. 29) prediction scores ≥0.957 (probably damaging variants). PolyPhen-2 predicts possible impact of an amino acid substitution on the structure and function of human proteins. In the first functional definition set, variant pruning for each gene was completed within each ethnic group by randomly selecting one variant for each pair of functional variants with LD *r*^2^>0.9 (*r*^2^ and *D*’ values were calculated with PLINK). Requiring each gene to comprise at least 3 functional variants, we considered a total of 140,986 functional coding variants (8,933 genes) in European Americans, 148,677 functional variants (10,342 genes) in African Americans, 149,236 functional variants (10,439 genes) in Latinos and 151,006 functional variants (9,534 genes) in the combined sample. In the second functional definition set, we considered a total of 20,077 functional coding variants (1,977 genes) in European Americans, 22,625 functional variants (2,465 genes) in African Americans, 22,644 functional variants (2,427 genes) in Latinos and 22,248 functional variants (2,316 genes) in the combined sample. We performed a meta-analysis of gene-based association tests applying the first five principal components as implemented in MetaSKAT-O^28^. Analyses were completed with two sets of variant weights: either larger weights on rare variants (MetaSKAT-O default parameter) or equal weights for all variants. Bonferroni significance thresholds were applied (*α*=0.05) to correct for the number of genes tested within each group and in the combined sample.

### Assessment of functional variants on the Illumina exome array

WGS for the 278 asthmatics was carried out at Complete Genomics (CGI) and resulted in an average read depth of 95.4% at 20 × or greater across the exome target and 91.4% across the genome. We excluded singletons from this analysis that were discovered in the 278 WGS but not present in dbSNP137 or in the Exome Sequencing Project variant server. We selected functional variants (missense, nonsense and splice site mutations) that were of low-frequency or rare (MAF <5%) in any of the ethnic groups in the WGS and identified the overlap with the variants present on the exome array.

## Additional information

**How to cite this article:** Igartua, C. *et al*. Ethnic-specific associations of rare and low-frequency DNA sequence variants with asthma. *Nat. Commun.* 6:5965 doi: 10.1038/ncomms6965 (2015).

## Supplementary Material

Supplementary Information Supplementary Figures 1-9, Supplementary Tables 1-6, Supplementary Notes 1-2 and Supplementary References

## Figures and Tables

**Table 1:** Sample composition.

**Sample**	**Case**	**Control**	**Male**	**Female**	**Total**
*European American*					
ALHS	776	802	859	719	1,578
CAG	186	209	172	223	395
CAMP*	376	706	588	494	1,082
CHS	415	640	546	509	1,055
COAST	134	119	150	103	253
Total	1,511376*	1,770	2,315	2,048	4,363
					
*African Ancestry*					
CAG	274	200	170	304	474
GRADD-AA	236	288	199	325	524
GRADD-AC†	227	297	250	274	524
SAPPHIRE	553	233	278	508	786
Total	1,290	1,018	897	1,411	2,308
					
*Latino*					
CHS	581	775	713	643	1,356
GALA II-MA	568	604	568	604	1,172
GALA II-PR	844	540	731	653	1,384
MCAAS*	214	428	337	305	642
Total	1,993214*	1,919	2,349	2,205	4,554
					
*Combined*					
Total	4,794590*	4,707	5,561	5,664	11,225

ALHS, Agricultural Lung Heath Study; CAG, Chicago Asthma Genetics Study; CAMP, Childhood Asthma Management Program; CHS, The Children’s Health Study; COAST, The Childhood Origins of Asthma Study; GALA II, Genes–Environment and Admixture in Latino Asthmatics (-MA, Mexican American; -PR, Puerto Rican); GRAAD, Genomic Research on Asthma in the African Diaspora and Barbados (-AA, African Americans; -AC, African Caribbeans); MCCAS, Mexico City Childhood Asthma Study; SAPPHIRE, The Study of Asthma Phenotypes and Pharmacogenomic Interactions by Race-Ethnicity.

Descriptions of the individual study samples have either been published^4^,^10^ or are described in Supplementary Note 1.

^*^Case–parent trios.

^†^Samples from 154 families; kinship matrix was used to address family structure as implemented in GEMMA^15^.

**Table 2:**
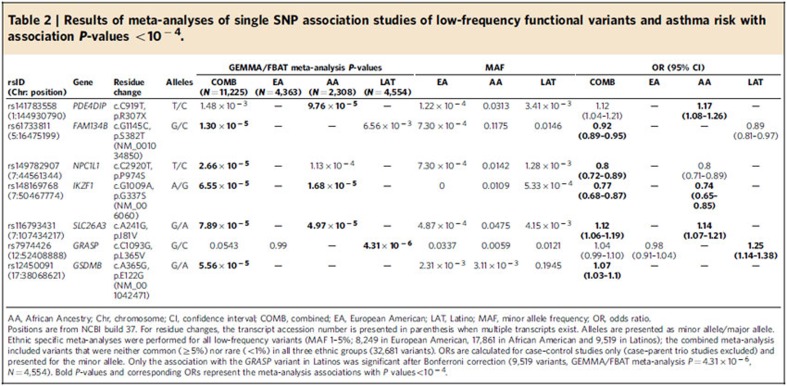
Results of meta-analyses of single SNP association studies of low-frequency functional variants and asthma risk with association *P*-values <10^−4^.

**Table 3:** Conditional analysis results for rs12450091 (c.A365G, p.E122G) in *GSDMB* after conditioning on 17q12–21 genotypes at rs7216389, rs2305480, and rs907092.

**rsID** **(Chr:Position)**	**Gene**	**Residue Change**	**Alleles**	**GEMMAmeta-analysis** ***P*** **(COMB)**	**r** ^2^ **/D′** **(LAT)**	**Conditional GEMMA meta-analysis** ***P*** **for rs12450091 (c.A365G, p.E122G)**	
						**COMB(no covariate** ***P*****=5.52 × 10**^**−5**^**)**	**LAT(no covariate** ***P*****=6.89** × **10**^−**5**^**)**
rs907092 (17:37922259)	* IKZF3 *	c.C1314T, p.S438S(NM_012481)	A/G	2.08 × 10^−10^	0.102/0.97	0.0190	0.0215
rs2305480 (17:38062196)	* GSDMB *	c.C892T, p.P298S(NM_001042471)	T/C	1.09 × 10^−8^	0.104/0.99	0.0139	0.0160
rs7216389 (17:38069949)	* GSDMB *	Intronic	C/T	1.23 × 10^−7^	0.13/1	0.0131	0.0149

Chr, chromosome; COMB, combined; LAT, Latino.

Three 17q12–21 common variants that were included on the array and associated with asthma in previous GWAS (rs907092, rs2305480, rs7216389) were included as covariates in association studies of rs12450091 in the case-control samples only (case-parent trio studies excluded and meta *P*-value recalculated). Conditional analyses included 3,912 subjects for the Latino study and 9,501 for the combined study. Results for rs12450091 with and without including the common variants are shown. Positions are from NCBI build 37. LD r^2^ and D’ between rs12450091 and each common variant were estimated in 4,696 unrelated Latino individuals in our study.

**Table 4:** Genes significantly associated with asthma.

**Sample**	**Gene**	**Chr**	**No. variants**	**MetaSKAT-O** ***P***
*a. Functional variants: missense, nonsense and splice site*				
Combined	* GSDMB *	17	16	6.10 × 10^−10^
Combined	* ZPBP2 *	17	7	1.34 × 10^−6^
Latino	* GSDMB *	17	12	1.02 × 10^−6^
Latino	* ZPBP2 *	17	7	1.73 × 10^−6^
African Ancestry	* MTHFR *	1	11	1.72 × 10^−6^
				
*b. Functional variants: PolyPhen-2 probably damaging variants*				
Combined	* GSDMB *	17	7	4.09 × 10^−8^
Latino	* GSDMB *	17	5	7.81 × 10^−8^

Chr, chromosome; No, number.

Gene-based analyses of functional variants were conducted using equal variant weights. All genes with at least 3 functional variants present in at least two studies were included in this analysis. Genes presented in the table are significant after Bonferroni correction for the number of genes tested. Meta-SKAT-O^28^ gene-based analyses included 3,281 subjects for the European American studies, 2,308 for the African ancestry studies, 3,912 subjects for the Latino studies and 9,501 subjects for the combined studies. a. All missense, nonsense and splice site variants (regardless of MAF) were considered, resulting in analysis of 8,933 genes in European Americans, 10,342 genes in African Ancestry groups, 10,439 genes in Latinos and 9,534 genes in the combined sample. b. Only variants that are predicted to be ‘probably damaging’ by PolyPhen-2 (ref. 29) were considered, resulting in analysis of 1,977 genes in European Americans, 2,465 genes in African Ancestry, 2,427 genes in Latinos and 2,316 genes in the combined sample. Variants included in the gene-based analyses are provided in Supplementary Table 3.

**Table 5:** Functional variant discovery in WGS of 278 asthmatics.

**Ethnic group**	**WGS sample size**	**No. functional variants in WGS**		**No. genes with functional variants in WGS**	
		**Total**	**Present on exome array (%)**	**Total**	**Present on exome array (%)**
European American	101	36,202	22,955 (63.40%)	12,568	10,044 (79.91%)
African Ancestry	93	59,851	38,227 (63.87%)	14,452	12,063 (83.47%)
Latino	84	37,308	23,420 (62.77%)	12,441	9,865 (79.29%)
Combined	278	105,943	65,170 (61.51%)	16,359	14,222 (86.93%)

No, number; WGS, whole genome sequences.

Functional variants in this analysis include all missense, nonsense and splice site mutations with MAF<5% within any of the three ethnic groups. Functional variants in the WGS were excluded if they were absent in dbSNP137 or in the ESP variant server.
