# Supplementary material for: Ethnic-specific associations of rare and low-frequency DNA sequence variants with asthma
Source: Nat Commun. 2015 Jan 16;6:5965. doi: 10.1038/ncomms6965 (PMC4309441; doi:10.1038/ncomms6965)
Supplement: Supplementary Information — Supplementary Figures 1-9, Supplementary Tables 1-6, Supplementary Notes 1-2 and Supplementary References [file ncomms6965-s1.pdf]

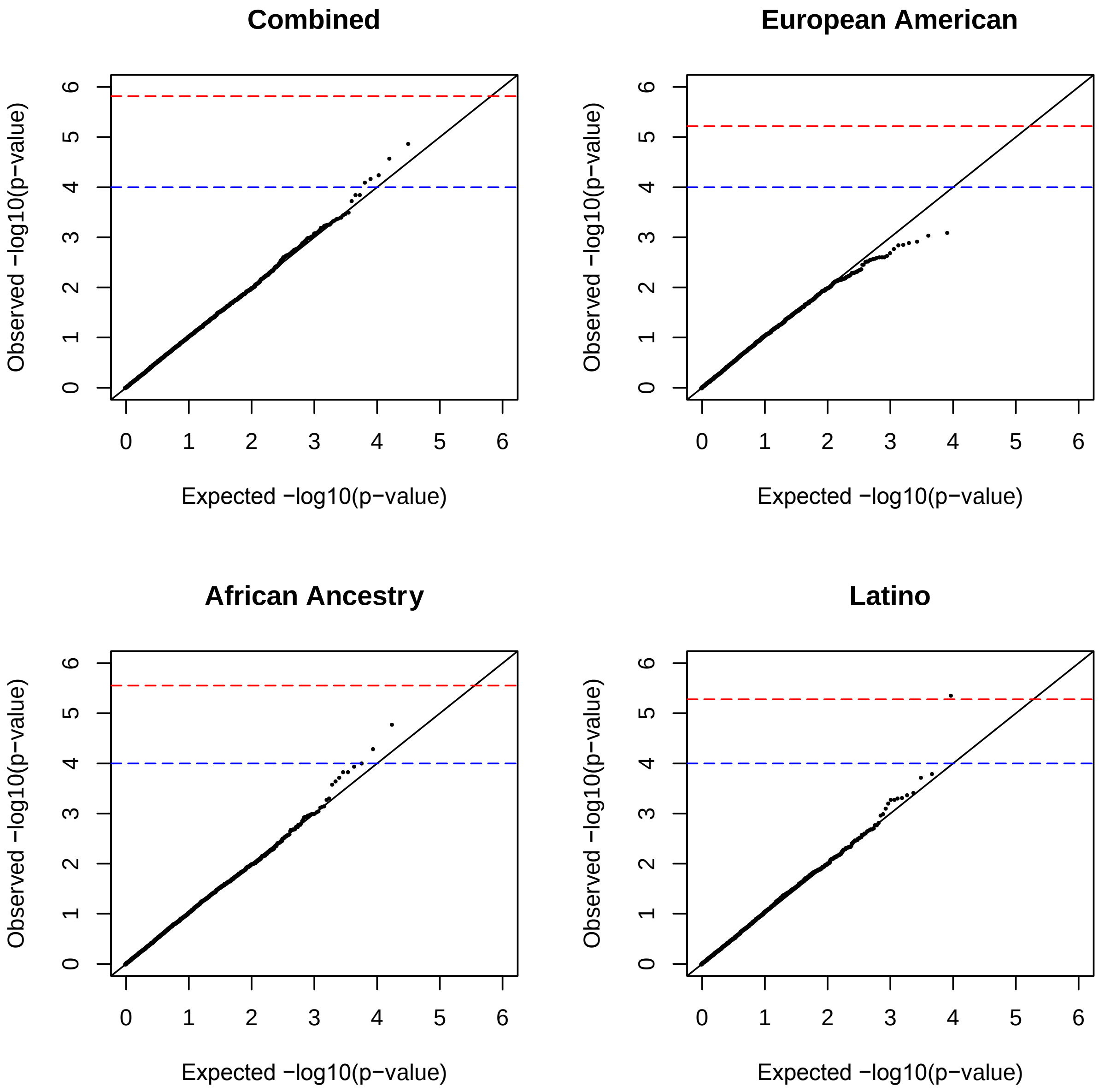

Supplementary Figure 1: QQplots of single variant meta-analyses of low frequency, functional variants and asthma risk. We considered 32,681 variants in the combined sample (N=11,225), 8,249 in European Americans (N=4,363), 17,861 in African Ancestry (N=2,308), and 9,519 in Latinos (N=4,554). Dashed red line shows the Bonferroni threshold of significance (corrected for number of variants tested). Dashed blue line shows *P*=0.0001.

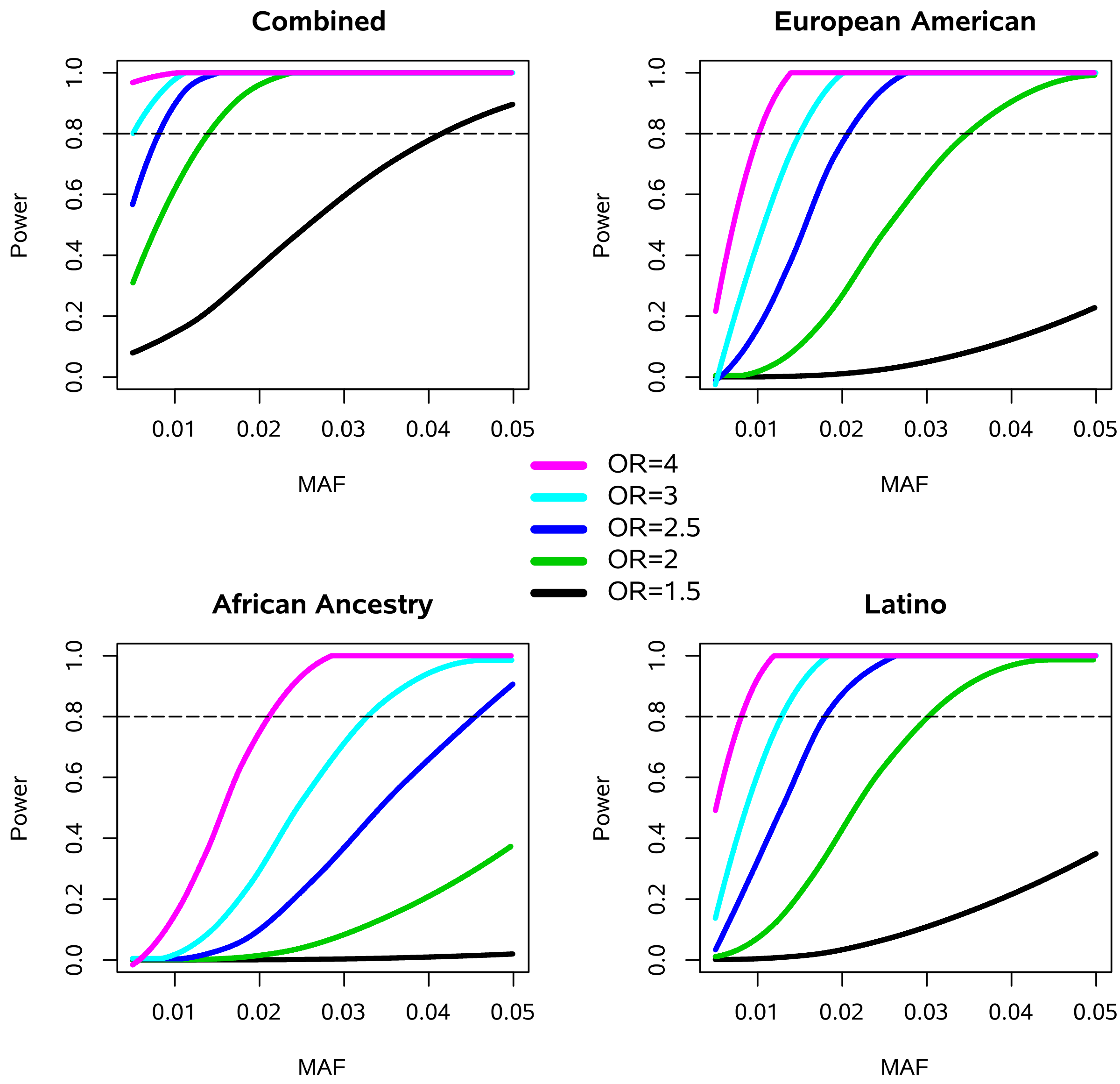

Supplementary Figure 2: Power analyses for low frequency single variant meta analyses. For each ethnic group and the combined sample, 1,000 variants with MAF 0.5%-5% were randomly sampled for simulations; meta-analyses were performed using the same allele frequency structure in our data and the same study design of our study to maintain a 5% type I error. We performed simulations with ORs=1.5, 2, 2.5, 3, and 4, and fit a local linear regression curve. Dashed horizontal lines represents 80% power in the meta-analysis.

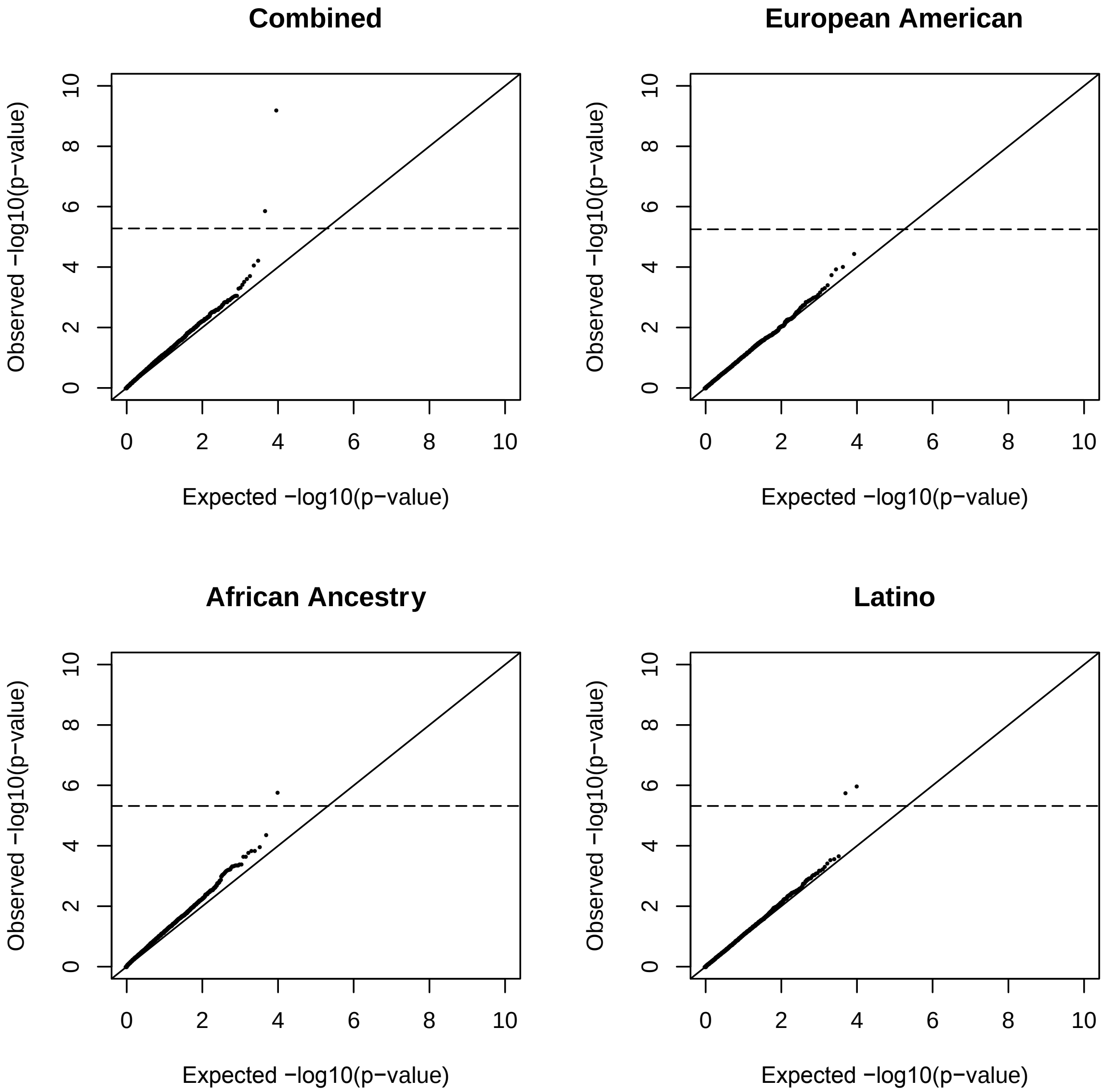

Supplementary Figure 3: Gene-based meta-analyses qqplots for functional variants defined as missense, nonsense and splicing and equal variant weights. Gene-based analyses of functional variants were conducted using equal variant weights (MetaSKAT-O[1]). All genes with at least 3 functional variants that were present in at least two studies were included in this analysis. All missense, nonsense and splice site variants (regardless of MAF) were considered functional, resulting in analysis of 8,933 genes in European Americans (N=3,281), 10,342 genes in African Americans (N=2,308), 10,439 genes in Latinos (N=3,912) and 9,534 genes in the combined sample (N=9,501). Dashed line represents the Bonferroni threshold of significance (corrected for number of genes tested).

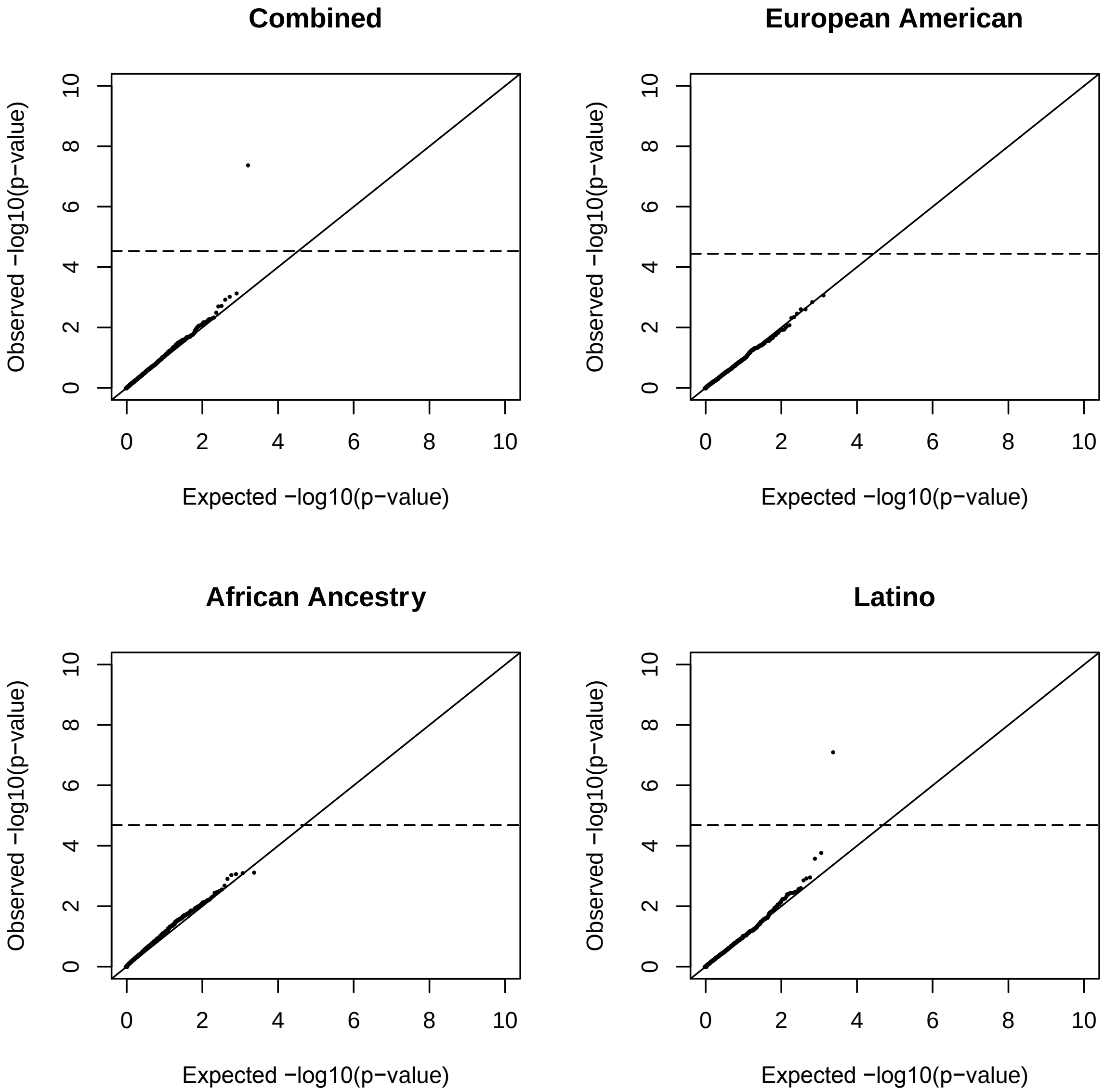

Supplementary Figure 4: Gene-based meta-analyses qqplots for functional variants defined as PolyPhen-2 probably damaging variants and equal variant weights. Gene-based analyses of functional variants were conducted using equal variant weights (MetaSKAT-O). All genes with at least 3 functional variants that were present in at least two studies were included in this analysis. All variants predicted to be 'probably damaging' by PolyPhen-2 were considered functional, resulting in analysis of 1,977 genes in European Americans (N=3,281), 2,465 genes in African Ancestry (N=2,308), 2,427 genes in Latinos (N=3,912) and 2,316 genes in the combined sample (N=9,501). Dashed line represents the Bonferroni threshold of significance (corrected for number of genes tested).

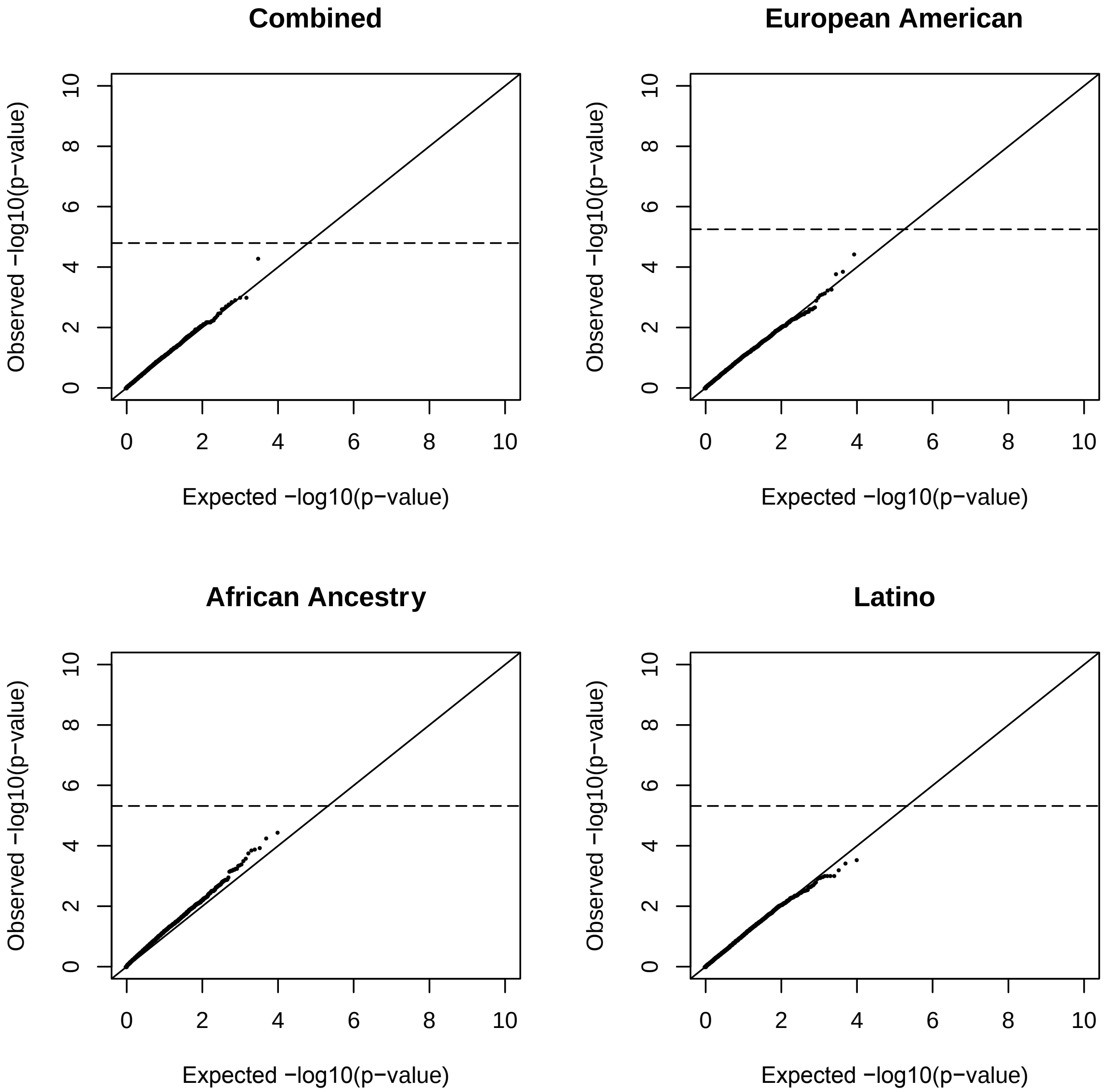

Supplementary Figure 5: Gene-based meta-analyses qqplots for functional variants defined as missense, nonsense and splicing and rare variant weights. Gene-based analyses of functional variants were conducted using rare variant weights (MetaSKAT-O default). All genes with at least 3 functional variants that were present in at least two studies were included in this analysis. All missense, nonsense and splice site variants (regardless of MAF) were considered functional, resulting in analysis of 8,933 genes in European Americans (N=3,281), 10,342 genes in African Americans (N=2,308), 10,439 genes in Latinos (N=3,912) and 9,534 genes in the combined sample (N=9,501). Dashed line represents the Bonferroni threshold of significance (corrected for number of genes tested).

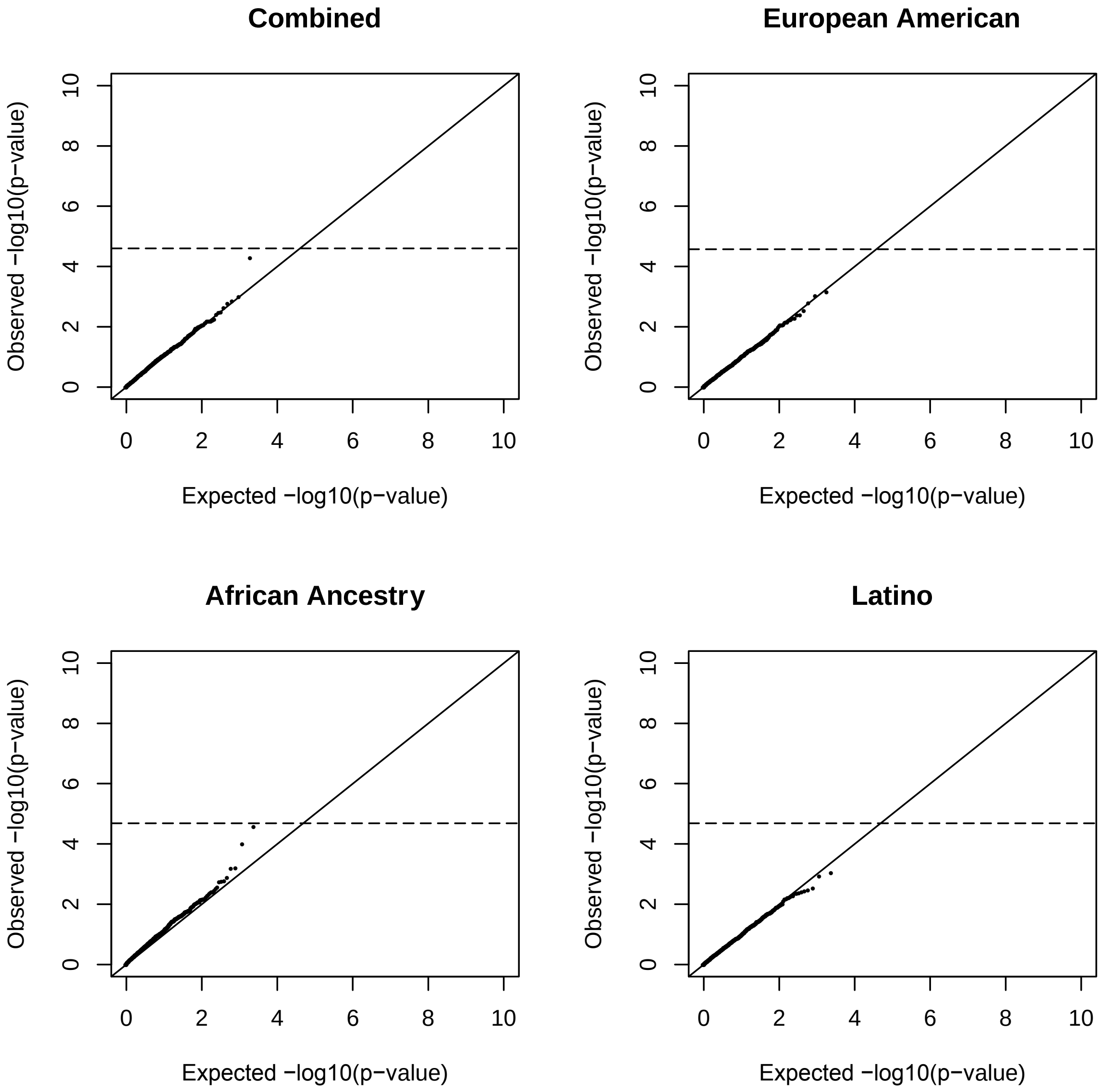

Supplementary Figure 6: Gene-based meta-analyses qqplots for functional variants defined as PolyPhen-2 probably damaging variants and rare variant weights. Gene-based analyses of functional variants were conducted using rare variant weights (MetaSKAT-O default). All genes with at least 3 functional variants that were present in at least two studies were included in this analysis. All variants predicted to be 'probably damaging' by PolyPhen-2 were considered functional, resulting in analysis of 1,977 genes in European Americans (N=3,281), 2,465 genes in African Ancestry (N=2,308), 2,427 genes in Latinos (N=3,912) and 2,316 genes in the combined sample (N=9,501). Dashed line represents the Bonferroni threshold of significance (corrected for number of genes tested).

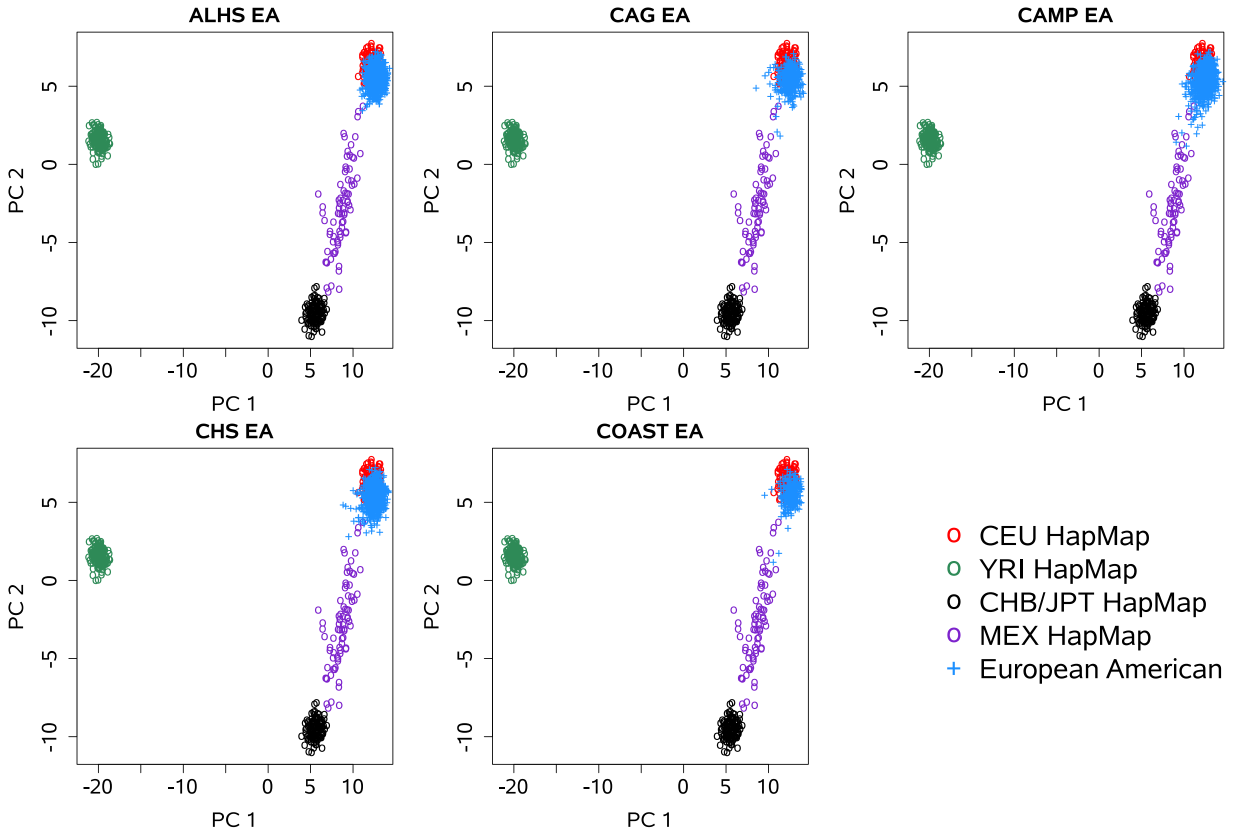

Supplementary Figure 7: PCA of European American studies projected onto HapMap subjects. Each panel represents one of the 5 European American studies included in the meta analysis[2,3] (ALHS, Agricultural Lung Heath Study[4] [Supplementary Note 1]; CAG, Chicago Asthma Genetics Study; CAMP, Childhood Asthma Management Program; CHS, The Children's Health Study; COAST, The Childhood Origins of Asthma Study). Open circles represent HapMap[5] subjects of known ethnicities (red, CEU; green, YRI; black, CHB/JPT; purple, MEX). Blue plus signs (+) represent European American study subjects.

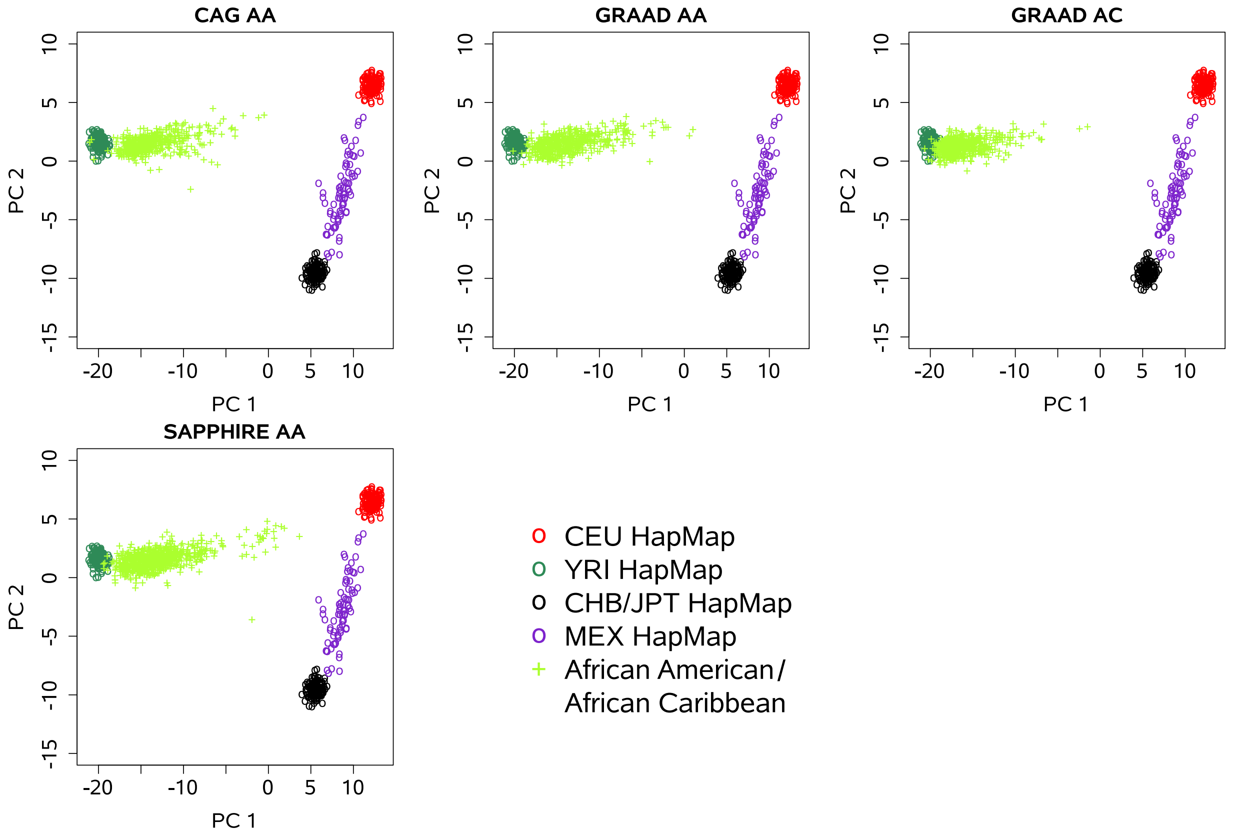

Supplementary Figure 8: PCA of African ancestry studies projected onto HapMap subjects. Each panel represents one of the 4 African ancestry studies included in the meta analysis[2,3] (CAG, Chicago Asthma Genetics Study; GRAAD, Genomic Research on Asthma in the African Diaspora and Barbados [-AA, African Americans; -AC, African Caribbeans]; SAPPHIRE, The Study of Asthma Phenotypes and Pharmacogenomic Interactions by Race-Ethnicity). Open circles represent HapMap subjects of known ethnicities (red, CEU; green, YRI; black, CHB/JPT; purple, MEX). Yellow-green plus signs (+) represent African ancestry study subjects.

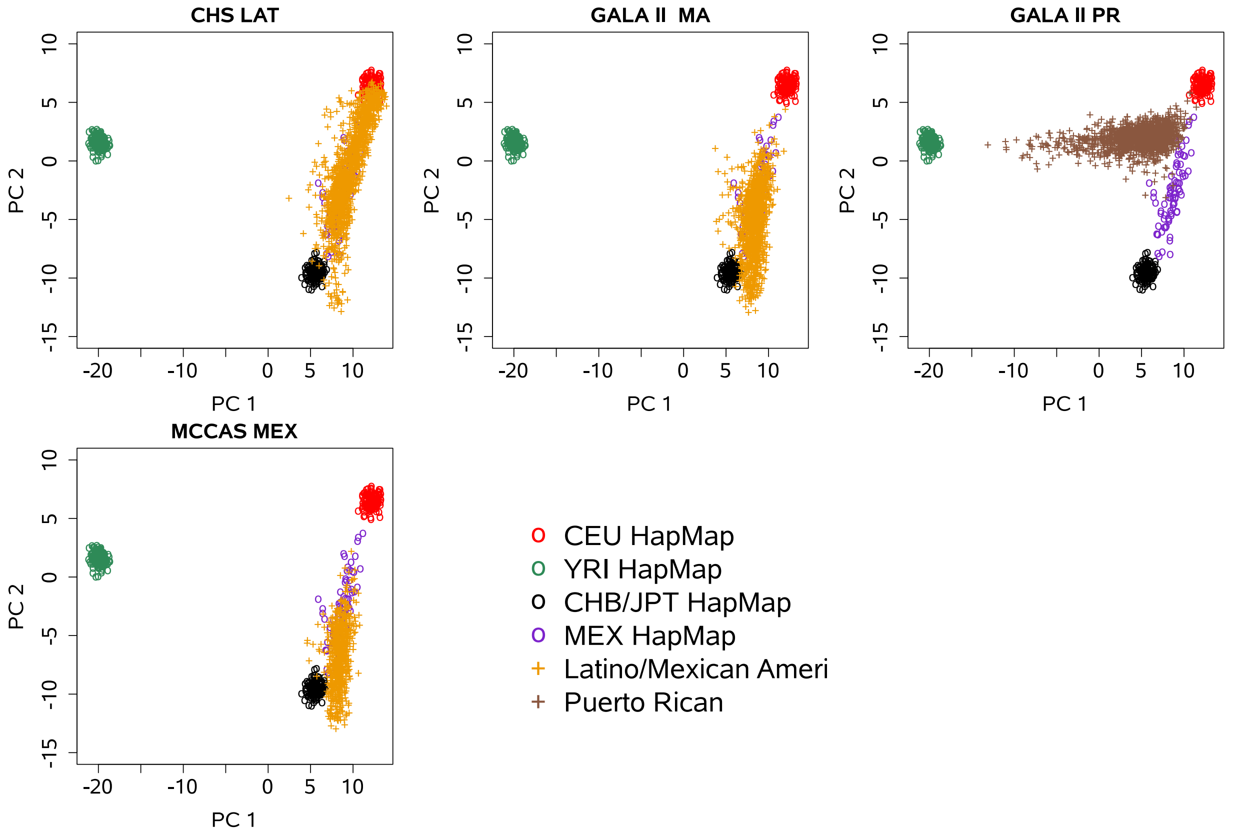

Supplementary Figure 9: PCA of Latino studies projected onto HapMap subjects. Each panel represents one of the 4 Latino studies included in the meta analysis[2,3] (CHS, The Children's Health Study; GALA II, Genes-Environment & Admixture in Latino Asthmatics [-MA, Mexican American; -PR, Puerto Rican]; MCCAS, Mexico City Childhood Asthma Study [-MEX, Mexican]). Open circles represent HapMap subjects of known ethnicities (red, CEU; green, YRI; black, CHB/JPT; purple, MEX). Orange and brown plus signs (+) represent Latino and Puerto Rican study subjects, respectively.

Supplementary Table 1: Number of variants on the exome array that passed QC and are variable in 11,225 study subjects.

Ethnic group	Total variants	Private variants	Common variants	Low-frequency variants	Rare variants
EA (N=4,363)	133,748	17,227	26,967	9,470	97,311
AA (N=2,308)	139,694	14,704	31,803	19,496	88,395
LAT (N=4,554)	159,189	13,559	28,005	10,667	120,517
Total (N=11,225)	197,339	-	23,206	36,413	137,720
Total Functional	171,677	-	9,459	32,681[a]	129,537

EA, European American; AA, African Ancestry; LAT, Latino.

[a]Variants included in single-variant meta-analyses.

Total counts for common and rare variants include SNPs with allele frequencies ≥5% or <1%, respectively, in all three ethic groups; the remaining variants are referred to as low frequency. Breakdown of low frequency variants by functional category is provided in Supplementary Table 2.

Supplementary Table 2: Variants included in single variant analyses.

		MAF (1-5%)		
Variant type	Total (N=11,225)	EA (N==4,363)	AA (N=2,308)	LAT (N=4,554)
Missense	32,157	8,095	17,606	9,350
Nonsense	296	84	159	83
Splice site	228	70	96	86
Total	32,681	8,249	17,861	9,519

MAF Minor allele frequency; EA, European American; AA, African Ancestry; LAT, Latino.

Supplementary Table 3: Variants included in the gene-based meta-analyses at associated genes

A. Functional variants: all missense, nonsense and splice site

Meta-analysis	Gene	Chr.	No. variants	P	Variants included	Location	Variant Type	Alleles	EA MAF	AA MAF	LAT MAF	PolyPhen-2 score
Combined	*GSDMB*	17	16	$6.10x10^{-10}$	rs150508589	38061072	c.C1205G p.S402C	C/G	0.0015	0.0007	0.0010	1
					rs16965388	38062139	c.C949T p.R317C	A/G	0.0006	0.1083	0.0169	0.27
					rs2305479	38062217	c.G871A p.G291R	T/C	0.4850	0.1620	0.3349	1
					rs35266519	38062390	c.G823A p.D275N	T/C	0.0102	0.0017	0.0020	0.002
					rs199530486	38062422	c.T791C p.L264P	G/A	0.0002	0	0	1
					rs35104165	38062503	c.A710G p.D237G	C/T	0.0385	0.0068	0.0254	0.987
					rs141418331	38062509	c.C704T p.S235L	A/G	0	0	0.0001	0.002
					rs199540130	38063233	c.T669G p.D223E	C/A	0	0.0013	0.0002	0.987
					rs11078928	38064469	c.662-2A>G (NM_001165959)	C/T	0.4456	0.1217	0.3052	0
					rs201723403	38066146	c.C439T p.R147X	A/G	0	0	0.0002	0
					rs12450091	38068621	c.C278T p.T93M	C/T	0.0023	0.0031	0.1945	0.002
					rs140714868	38068708	c.A233G p.Q78R	A/G	0.0002	0.0015	0	0.69
					rs142509403	38073337	c.C180A p.D60E	C/T	0.0007	0	0	0.5
					rs150941805	38073390	c.G34T p.V12L	T/G	0.0009	0.0002	0.0002	1
					rs150365108	38073536	c.C278T p.T93M	A/C	0	0.0002	0	0.977
					rs138635056	38073569	c.A1G p.M1V	C/T	0	0.0039	0.0002	0.99
Combined	*ZPBP2*	17	7	$1.34x10^{-6}$	rs35591738	38027030	c.C202G p.P68A	G/C	0.0094	0.0013	0.0116	1
					rs35829084	38027824	c.G352A p.A118T	A/G	0.0002	0.1025	0.0077	0.155
					rs114337101	38028598	c.A482G p.D161G	G/A	0	0	0.0001	-
					rs11557467	38028634	c.G518T p.S173I	T/G	0.4933	0.3924	0.3576	0.295
					rs138757022	38031564	c.T766A p.F256I	A/T	0	0.0013	0.0001	0.201
					rs115778431	38031648	c.A850G p.K284E	G/A	0.0039	0.0013	0.0012	1
					rs35302660	38033048	c.C1003G p.Q335E	G/C	0.0009	0.1221	0.0191	0.017
Latino	*GSDMB*	17	12	$1.02x10^{-6}$	rs150508589	38061072	c.C1205G p.S402C	C/G	0.0016	0.0006	0.0010	1
					rs16965388	38062139	c.C949T p.R317C	A/G	0.0006	0.1083	0.0169	0.27
					rs2305479	38062217	c.G871A p.G291R	T/C	0.4850	0.162	0.3349	1
					rs35266519	38062390	c.G823A p.D275N	T/C	0.0102	0.0017	0.0020	0.002
					rs35104165	38062503	c.A710G p.D237G	C/T	0.0385	0.0068	0.0254	0.987

					rs141418331	38062509	c.C704T p.S235L	A/G	0	0	0.0001	0.002
					rs199540130	38063233	c.T669G p.D223E	C/A	0	0.0013	0.0002	0.987
					rs11078928	38064469	c.662-2A>G (NM_001165959)	C/T	0.4456	0.1217	0.3052	0
					rs201723403	38066146	c.C439T p.R147X	A/G	0	0	0.0002	0
					rs12450091	38068621	c.A365G p.E122G	C/T	0.0023	0.0031	0.1945	0.002
					rs150941805	38073390	c.C180A p.D60E	T/G	0.0008	0.0002	0.0002	1
					rs138635056	38073569	c.A1G p.M1V	C/T	0	0.0039	0.0002	0.99
Latino	*ZPBP2*	17	7	$1.73x10^{-6}$	rs35591738	38027030	c.C202G p.P68A	G/C	0.0093	0.0013	0.0116	1
					rs35829084	38027824	c.G352A p.A118T	A/G	0.0002	0.1025	0.0077	0.155
					rs114337101	38028598	c.A482G p.D161G	G/A	0	0	0.0001	-
					rs11557467	38028634	c.G518T p.S173I	T/G	0.4933	0.3924	0.3576	0.295
					rs138757022	38031564	c.T766A p.F256I	A/T	0	0.0013	0.0001	0.201
					rs115778431	38031648	c.A850G p.K284E	G/A	0.0038	0.0013	0.0012	1
					rs35302660	38033048	c.C1003G p.Q335E	G/C	0.0008	0.1221	0.0191	0.017
African Ancestry	*MTHFR*	1	11	$1.72x10^{-6}$	rs35737219	11850750	c.C1958T p.T653M	A/G	0.0245	0.0033	0.0126	0.002
					rs2274976	11850927	c.G1781A p.R594Q	T/C	0.0502	0.0266	0.0411	0
					rs45449298	11852411	c.G1556T p.R519L	A/C	0.0001	0.0042	0.0002	0.422
					rs45496998	11852412	c.C1555T p.R519C	A/G	0.0003	0.0066	0.00181	0.997
					rs144594875	11854076	c.G1418A p.R473Q	T/C	0	0.0015	0	0.797
					rs139645527	11854086	c.G1408C p.E470Q	G/C	0.0029	0.0002	0.0009	0.004
					rs143466425	11855279	c.G907A p.V303M	T/C	0	0.0002	0	0.999
					rs142612062	11855398	c.A788C p.H263P	G/T	0	0.0011	0.0001	0.283
					rs150847674	11856376	c.G667A p.D223	T/C	0	0.0042	0.0001	0.702
					rs1801133	11856378	c.C665T p.A222V	A/G	0.3348	0.1105	0.449	0.999
					rs149514973	11861298	c.G395A p.R132H	T/C	0	0.0004	0	0

B. Functional variants: PolyPhen-2 probably damaging variants only

Meta-analysis	Gene	Chr.	No. variants	P	Variants included	Location	Variant Type	Alleles	EA MAF	AA MAF	LAT MAF	PolyPhen-2 score
Combined	*GSDMB*	17	7	$4.09x10^{-8}$	rs2305479	38062217	c.G871A p.G291R	T/C	0.4850	0.1620	0.3349	1
					rs199530486	38062422	c.T791C p.L264P	G/A	0.0002	0.0000	0.0000	1
					rs35104165	38062503	c.A710G p.D237G	C/T	0.0386	0.0069	0.0255	0.987

					rs199540130	38063233	c.T669G p.D223E	C/A	0.0000	0.0013	0.0002	0.987
					rs150941805	38073390	c.C180A p.D60E	T/G	0.0009	0.0002	0.0002	1
					rs150365108	38073536	c.G34T p.V12L	A/C	0.0000	0.0002	0.0000	0.977
					rs138635056	38073569	c.A1G p.M1V	C/T	0.0000	0.0040	0.0002	0.99
Latino	*GSDMB*	17	5	7.81×10^{-8}	rs2305479	38062217	c.G871A p.G291R	T/C	0.4850	0.1620	0.3349	1
					rs35104165	38062503	c.A710G p.D237G	C/T	0.0386	0.0069	0.0255	0.987
					rs199540130	38063233	c.T669G p.D223E	C/A	0.0000	0.0013	0.0002	0.987
					rs150941805	38073390	c.C180A p.D60E	T/G	0.0009	0.0002	0.0002	1
					rs138635056	38073569	c.A1G p.M1V	C/T	0.0000	0.0040	0.0002	0.99

Chr, chromosome; No, number; EA, European American; AA, African Ancestry; LAT, Latino; MAF Minor allele frequency.

Positions are from NCBI build 37; Alleles presented as minor allele/major allele.

Variant type presented for the following transcript accessions unless otherwise noted: *GSDMB* (NM_001042471), *ZPBP2* (NM_199321), *MTHFR* (NM_005957).

PolyPhen-2 scores predict the impact of an amino acid substitution on the structure and function of a human protein. Scores ≥0.957 are predicted to be 'probably damaging'.

Gene-based analyses of functional variants were conducted using equal variant weights. All genes with at least 3 functional variants present in at least two studies were included in this analysis. Genes presented in the table are significant after Bonferroni correction for the number of genes tested. Meta-SKAT-O gene-based analyses included 3,281 subjects for the European American studies, 2,308 for the African ancestry studies, 3,912 subjects for the Latino studies and 9,501 subjects for the combined studies. **A.** All missense, nonsense and splice site variants (regardless of MAF) were considered resulting in analysis of 8,933 genes in European Americans, 10,342 genes in African Ancestry groups, 10,439 genes in Latinos and 9,534 genes in the combined sample. **B.** Only variants that are predicted to be 'probably damaging' by PolyPhen-2[6] were considered resulting in analysis of 1,977 genes in European Americans, 2,465 genes in African Ancestry, 2,427 genes in Latinos and 2,316 genes in the combined sample.

Supplementary Table 4: Representation of genes on the exome array with functional variants in the whole genome sequences of 278 asthmatic individuals.

Ethnic group	No. genes not represented on the array	No. genes represented on the array but not all WGS variants included	No. genes represented on the array and all WGS functional variants included
EA	2,524 (20.08%)	4,604 (36.63%)	5,440 (43.28%)
AA	2,389 (16.53%)	7,198 (49.80%)	4,865 (33.66%)
LAT	2,576 (20.70%)	4,729 (38.01%)	5,136 (41,28%)
COMB	2,137 (13.06%)	10,686 (65.32%)	3,536 (21.61%)

No, number; WGS, whole genome sequence; EA, European American; AA, African American; LAT, Latino; COMB, combined.

Supplementary Table 5: Exome array variants removed from analysis.

Quality control exclusion category	Probes removed
Call rate <95%	2,280
SNPs not variable in our study	47,455
European American HWE	229
African American HWE	385
African Caribbean HWE	138
Mexican-American HWE	527
Puerto Rican HWE	242
Mitochondria SNPs	163
Caution site	333
Total unique number of SNPs removed	50,437

HWE, Hardy-Weinberg equilibrium.

Supplementary Table 6: Total subject exclusions.

Exclusion Category	No. Subjects
Gender discordancy	27
Sample duplication[a]	107
Ancestry discordancy[b]	56
Mendelian error[c]	9
Missing case/control status	7
Incomplete trios[c]	473
Total subjects removed	679

No, Number;

[a]Sample with lowest genotype call rate excluded.

[b]Sample excluded if either of the top two inferred principle component was more than six standard deviations from the mean.

[c]Case-parent trios only.

Supplementary Note 1: Description of the Agricultural Lung Health Study.

The Agricultural Lung Health Study (ALHS) is a case-cohort study nested within the larger Agricultural Health Study (AHS)[4]. The AHS includes 52,394 subjects who are private pesticide applicators (mainly farmers) in North Carolina and Iowa and 32,345 spouses of farmers. Applicators enrolled in the study from 1993 to 1997 at pesticide licensing facilities by completing self-administered questionnaires. Subjects have been followed up by questionnaires. ALHS recruited participants based on their response to questions about current asthma and asthma symptoms reported during the AHS telephone follow-up interview conducted between 2005-2010. Current asthma cases who were selected for screening were defined as 1) presumptive current asthma (doctor diagnosed asthma with current symptoms or medication use, 81%) or 2) presumptive undiagnosed asthma (persistent wheeze or inhaler use for asthmatic symptoms, 19%). A random cohort sample was also generated from among respondents to the 2005-2010 telephone interview. DNA was extracted from blood for most subjects (approximately 95%), with the remainder from buccal cells collected using Oragene kits (DNA Genotek, Kanata, Ontario, Canada). Participants in the current study were subjects who self-reported as Caucasian enrolled from February 2009 through August 2011. Genotyping for the Illumina HumanExome BeadChip was conducted at the Genetic Resources Core Facility, Johns Hopkins Institute of Genetic Medicine, Baltimore, MD. From among 1,653 subjects genotyped, 31 failed quality control (3 sex discordances and 28 investigator reported exclusions), 44 were not of Caucasian American based on principal components analysis leaving 1,578 subjects for analysis including 776 cases and 802 controls.

Supplementary Note 2: Additional probe and genotyping quality evaluation.

To further evaluate probe quality in the exome array and genotype calling, genotypes in the 7,879 EVE samples were re-called using optiCall[7]. Out of the SNPs that had passed QC filters, 5,244 SNPs (2.15%) resulted in optiCall call rates below 95% and 1,908 SNPs (0.78%) had concordance rates less than 99% between Illumina's genotype caller (GenCall) and optiCall (overall 6,302 unique SNPs). In addition, 82 of the 278 asthmatics with WGS were also genotyped on the exome array platform (including only samples genotyped at the NWGC). Genotype concordance rates for these 82 individuals were calculated for each of the two genotype callers and SNPs were flagged if 5 or more samples (6%) were identified as discordant. GenCall resulted in 3,079 (1.24%) discordant SNPs while optiCall had 3,567 SNPs (1.43%). Based on the slightly higher concordance rate for GenCall, we selected to continue with the original Illumina genotype caller algorithm.

Supplementary References:

1. Lee, S., Teslovich, T. M., Boehnke, M. & Lin, X. General framework for meta-analysis of rare variants in sequencing association studies. *Am. J. Hum. Genet.* **93,** 42–53 (2013).
2. Torgerson, D. G. *et al.* Meta-analysis of genome-wide association studies of asthma in ethnically diverse North American populations. *Nat. Genet.* **43,** 887–892 (2011).
3. Myers, R. A. *et al.* Further replication studies of the EVE Consortium meta-analysis identifies 2 asthma risk loci in European Americans. *J. Allergy Clin. Immunol.* **130,** 1294–1301 (2012).
4. Alavanja, M. C. *et al.* The Agricultural Health Study. *Environ. Health Perspect.* **104,** 362–369 (1996).
5. Gibbs, R. A. *et al.* The International HapMap Project. *Nature* **426,** 789–796 (2003).
6. Adzhubei, I. A. *et al.* A method and server for predicting damaging missense mutations. *Nat. Methods* **7,** 248–249 (2010).
7. Shah, T. S. *et al.* optiCall: a robust genotype-calling algorithm for rare, low-frequency and common variants. *Bioinformatics* **28,** 1598–1603 (2012).
